# Synthesis methods of 1,2,3-/1,2,4-triazoles: A review

**DOI:** 10.3389/fchem.2022.891484

**Published:** 2022-09-26

**Authors:** Jinlian Dai, Sen Tian, Xueqing Yang, Zongliang Liu

**Affiliations:** School of Pharmacy, Key Laboratory of Molecular Pharmacology and Drug Evaluation (Yantai University), Ministry of Education, Collaborative Innovation Center of Advanced Drug Delivery System and Biotech Drugs in Universities of Shandong, Yantai University, Yantai, China

**Keywords:** 1,2,3-triazole, 1,2,4-triazole, synthesis method, heterocycle, derivatives

## Abstract

Triazole, comprising three nitrogen atoms and two carbon atoms, is divided into two isomers 1,2,3-triazole and 1,2,4-triazole. Compounds containing a triazole are one of the significant heterocycles that exhibit broad biological activities, such as antimicrobial, analgesic, anti-inflammatory, anticonvulsant, antineoplastic, antimalarial, antiviral, antiproliferative, and anticancer activities. A great quantity of drugs with a triazole structure has been developed and proved, for example, ketoconazole and fluconazole. Given the importance of the triazole scaffold, its synthesis has attracted much attention. This review summarizes the synthetic methods of triazole compounds from various nitrogen sources in the past 20 years.

## Introduction

Triazole is an important nitrogen heterocyclic compound, containing three nitrogen atoms and two carbon atoms. It had reported that compounds containing triazole have an important application value in various fields, such as (Brüggemann et al., 2022; Fallah et al., 2022; Sharma et al., 2022) agrochemistry (Georgiadis et al., 2018; Musarurwa and Tavengwa, 2021; Valencia-Quintana et al., 2022) and material chemistry (Scattergood et al., 2017; Rodrigues et al., 2020; Ahmed and Xiong, 2021). Its unique structure facilitates the formation of a variety of non-covalent bonds with enzymes and receptors inducing broad-spectrum biological activities, such as anticancer (Sharma et al., 2022), antituberculosis (Keri et al., 2015; Zhang et al., 2017), antibacterial (Gao et al., 2019; Zhang, 2019; Strzelecka and Swiatek, 2021), and anti-HIV (Patel et al., 2012).

**FIGURE 1:**
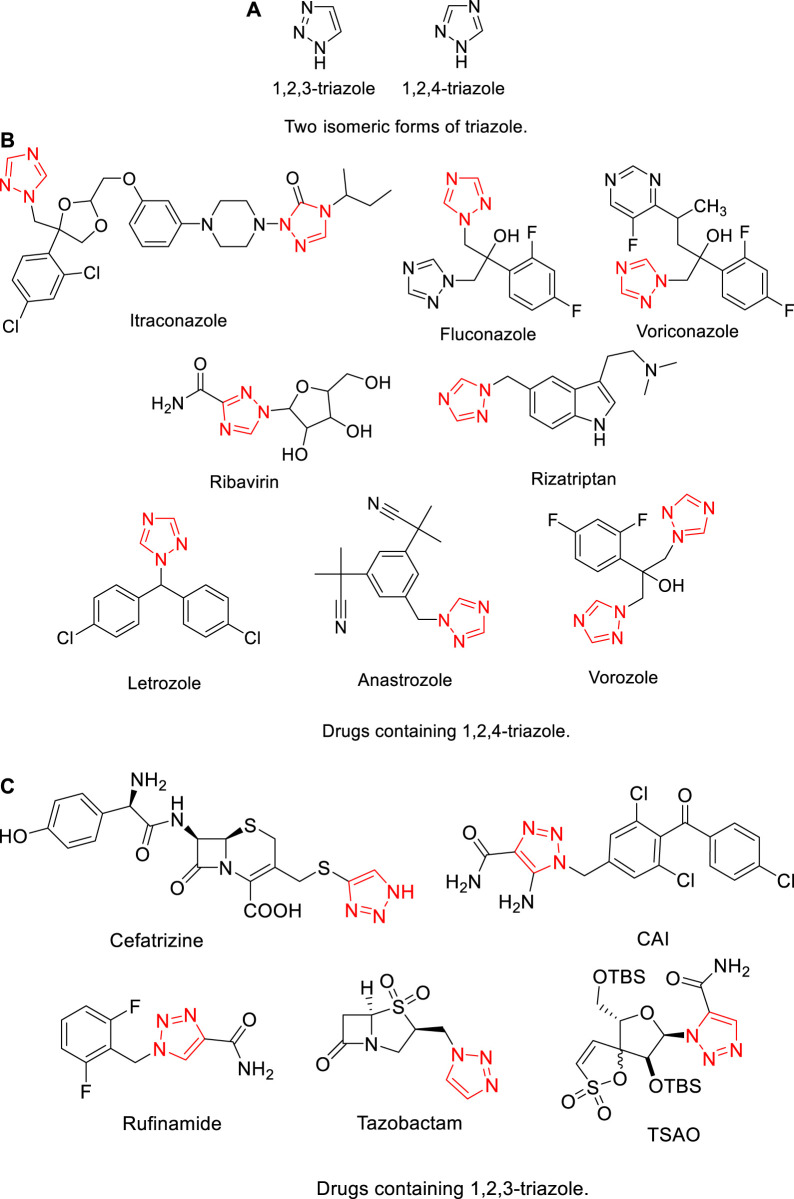
Triazole and drugs containing triazole.

**FIGURE 2:**
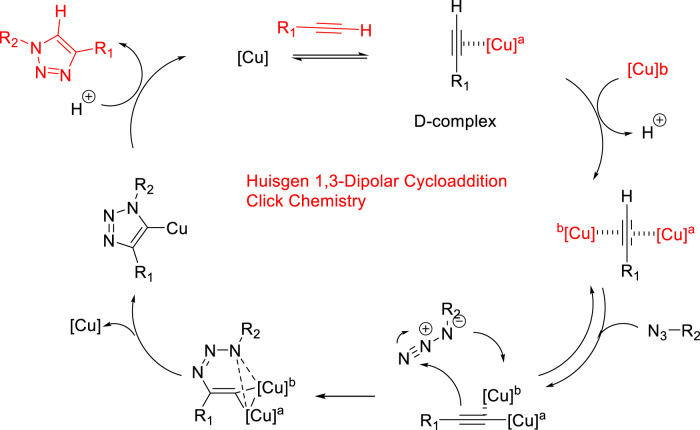
Cu-catalyzed azide–alkyne cycloaddition.

There are two isomeric forms of triazole, namely, 1,2,3-triazole and 1,2,4-triazole (Figure 1A). Their derivatives have been widely applied in many medicinal scaffolds (Figure 1;2 and (c)). For example, Itraconazole (Navarro-Triviño, 2021; Neto et al., 2022), Fluconazole (Elzoheiry et al., 2022; Gao et al., 2022), and Voriconazole (Shettar et al., 2021; Lindsay et al., 2022) are commonly used antifungals in clinical application. Ribavirin (Burman et al., 2022; Tian et al., 2022) is a broad-spectrum antiviral drug used in the treatment of hepatitis. Rizatriptan (Moore, 2007; Chokshi et al., 2019) has been incorporated into drug candidates as antimigraine agents. Letrozole (Park et al., 2021; Slomovitz et al., 2022), anastrozole (Kucukguzel and Cikla-Suzgun, 2015), and vorozole (Kim et al., 2009; Takahashi et al., 2011) as anticancer drugs are very effective. In addition, CAI and cefatrizine (Lucht et al., 1996) are very successful as anticancer drugs. Rufinamide (Caraballo et al., 2020; Sharawat et al., 2021) was approved by FDA for the treatment of pediatric epilepsy. Tazobactam (Pfaller et al., 2021; Ramassamy et al., 2021) has been successfully used in antibiotic therapy. TSAO (Dheer et al., 2017) is used in inhibition of HIV reverse transcriptase.

Given the importance of the triazoles, their synthesis has attracted much attention. Under thermal conditions, 1,3-dipolar cycloaddition between azide and terminal alkyne (also known as Watson cycloaddition) is the most popular reaction, which is suitable for producing 1,2,3-triazole moiety (Di Antonio et al., 2012) (Figure 2). However, it was only used to prepare 1,2,3-trizole in early stage due to weak regioselectivity (1,4- or 1,5-disubstituted-1,2,3-triazole), bad chemical yield, and high temperature. This method was reported at the start of the 20th century, but its specific mechanism was described by Huisgen et al. (1967). The click chemistry method of the Cu-catalyzed azide–alkyne cycloaddition reaction (CuAAC) proposed by Sharples can synthesize 1,2,3-triazole in high yield (Kolb et al., 2001) (Figure 2). As a major example of click chemistry, the CuAAC reaction has a great impact on organic synthesis and widely used in industry and academia. In view of the important application of triazole scaffolds in pharmaceutical intermediates, it is very significant to find a more effective synthetic method. Others reviews published successively in recent years about 1,2,3- or 1,2,4-triazole compounds, which focused on summarizing the structure–activity relationships (SAR) and synthetic strategy (Matin et al., 2022; Moura and Tomé, 2022; Nemallapudi et al., 2022). In addition, there are also some reviews focused on the synthesis of mono-/bis-aminomercapto and fused 1,2,4-triazole derivatives (Gomha et al., 2015; Riyadh and Gomha, 2020; Riyadh et al., 2022). In this review, combined with the progress of triazole synthesis in past 20 years, several main synthetic methods from various nitrogen sources are summarized. The synthetic methods were focused on the key starting material used for the production of the 1,2,3-/1,2,4-triazole skeleton.

### Synthesis of 1,2,3-triazoles

1,2,3-Triazole has strong stability for thermal and acid condition; otherwise, it is insensitive to redox, hydrolysis, and enzymatic hydrolase (Motornov et al., 2018). The main synthetic methods include sodium azido, trimethylsilyl azido, alkyl/aryl azido, hydrazone, sulfonamide, hydrazine, and diazo-compounds used as raw materials to provide nitrogen atoms for 1,2,3-triazole derivatives.

### Sodium azide as nitrogen source

**SCHEME 1:**
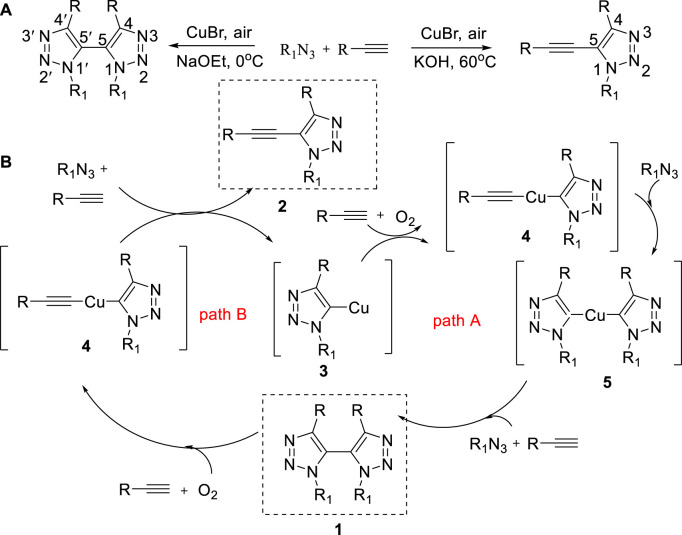
**(A)** Synthesis of bis(1,2,3-triazole) and 5-alkynyl-1,2,3-triazole by temperature regulation. **(B)** Possible mechanism of synthesis of bis(1,2,3-triazole) and 5-alkynyl-1,2,3-triazole.

In 2013, a simple method to synthesize bis(1,2,3-triazole) and 5-alkynyl-1,2,3-triazole from alkyne and azido at various temperatures was reported (Li et al., 2013). Various azidoes and alkynes as substrates participated in reactions, which had been successfully used in synthesis of bioactive nucleoside analogues. In this method, based on the temperature effect, copper bromide is used as a catalyst in the absence of a ligand. Between 0^o^C and 70°C, the yield of bis(1,2,3-triazole) increased with the decrease of reaction temperature and reached 91% (Li et al., 2013) after stirring for 20 h at 0°C. The opposite phenomenon: the yield of 5-alkynyl-1,2,3-triazole was highest (68%) at 60°C. As the temperature increases, the yield increases, and 60°C is the optimum temperature (Scheme 1A).

A possible mechanism of the temperature-mediated reaction is described in Scheme 1B. The triazolyl–copper complex intermediate 3 was generated by the CuAAC reaction. The tandem aerobic oxidative coupling reaction might be the key of this reaction. In addition, temperature might affect the reactivity of the triazolyl–copper complex 4 and 5. At 0°C, the aerobic oxidative coupling reaction could react preferentially by Path A to construct bis(1,2,3-triazole) 1. Meanwhile, at 60°C, the aerobic oxidative coupling reaction could react preferentially by Path B to construct 5-alkynyl-1,2,3-triazole 2.

**SCHEME 2:**
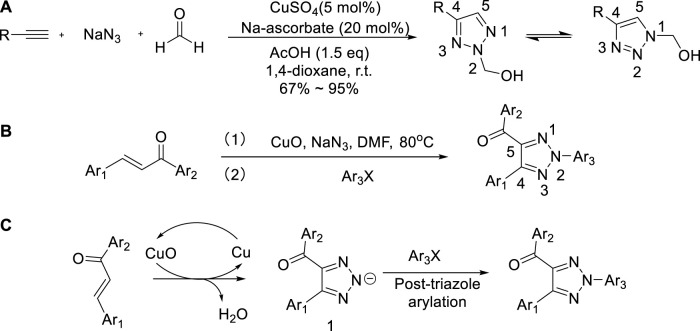
**(A)** Synthesis of 1,4- or 2,4-disubstituted-1,2,3-triazoles. **(B)** “One pot” three components synthesis of N2-substituted-1,2,3-triazole. **(C)** Possible mechanism of synthesis of the framework (b).

In 2008, Kalisiak reported an efficient three-component synthetic pathway for the production of 2-hydroxymethyl-2H-1,2,3-triazoles through Cu-catalyzed cycloaddition of terminal alkyne, NaN_3_, and formaldehyde in one pot (Jarosław Kalisiak and Fokin, 2008; Tian et al., 2022). Using copper as a catalyst, acetic acid was added in equal amounts and 1,4-dioxane was used as the solvent. At room temperature, yields ranged from 67% to 95% (Jarosław Kalisiak and Fokin, 2008; Tian et al., 2022). Due to the advantages of wide range of substrates and high compatibility of functional groups, the target products could be diversified (Scheme 2A).

In 2012, the “one pot” three components preparation of N2-substituted-1,2,3-triazole with chalcone, sodium azide, and halogenated aromatics as raw materials through cuprous oxide was proposed by Chen et al. (Zhang et al., 2012). The method could prepare selectively N2-substituted-1,2,3-triazoles and yield achieved was 98% (Zhang et al., 2012). It was an efficient strategy for multiple component condensation sequences about the combination of azide–chalcone oxidative cycloaddition and post-triazole arylation presented in diversity-oriented Scheme 2B. The mechanism study showed that cuprous oxide was used as an oxidant and a primer to accelerate the reaction. Corresponding products were obtained *via* nitrogen anion intermediates arylated with halogenated aromatics (Scheme 2C).

### Trimethylsilyl azide as nitrogen source

**SCHEME 3:**
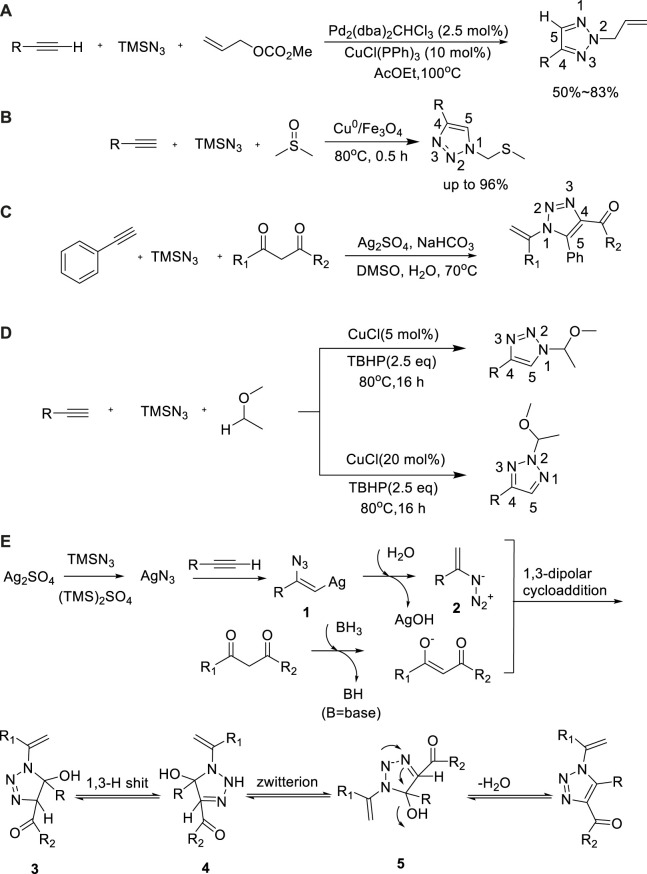
Synthesis and mechanism of triazole derivatives from TMSN_3_.

As an important nitride, trimethylsilyl azide is often used to synthesize various nitrogen-containing compounds. In 2003, Shin Kamijo used palladium/copper as a bimetallic catalyst to prepare 1,2,3-triazole based on the three-component cycloaddition reaction of alkynes, allyl methyl carbonate, and TMSN_3_ (Shin Kamijo et al., 2003). Using ethyl acetate as a solvent, 2-allyl-substituted-1,2,3-triazoles were obtained with moderate yield (56%–75%)(Shin Kamijo et al., 2003) at 100°C (Scheme 3A).

In 2016, another three-component reaction of alkynes, TMSN_3_, and dimethyl sulfoxide for synthesis of 1,2,3-triazole was developed (Phanindrudu et al., 2016). Kumar Tiwari et al. used nano-Cu^0^/Fe_3_O_4_ as an efficient catalyst for the regioselective synthesis of 1,2,3-triazoles by one-pot tandem; the yield was up to 96% (Phanindrudu et al., 2016). The reaction could be compatible with a variety of alkynes such as aromatic, aliphatic, heteraromatic, and polyaromatic compounds. The advantages of wide range of substrates and simple operation provided an attractive and convenient route for the synthesis of thiotriazole (Scheme 3B).

In 2019, Min Zhang et al. extended the substrate and employed silver sulfate as a catalyst, which showed the preparation of N1-vinyl-1,2,3-triazole by a three-component reaction of alkynes, TMSN_3_, and 1,3-diketone in high yield (82%)(Chen et al., 2019). N1-vinyl-1,2,3-triazoles were obtained using NaHCO_3_ as a base at 70°C. This method was simple to operate and had good compatibility with functional groups of substrates, providing an effective synthesis method for N1-vinyl-1,2,3-triazoles (Scheme 3C).

In 2019, Pengli Bao developed a similar reaction condition for selective production of 1,4- and 2,4-disubstitued-1,2,3-triazoles reaction of three-component of alkynes, TMSN_3_, and ethers by copper catalysis (Bao et al., 2019). The target products were obtained in high yield (86%) (Bao et al., 2019) at 80°C using tert-butanol peroxide as an oxidant and ether as a solvent and reactant. In addition, the synthesis of 1,4- and 2,4- disubstitued-1,2,3-triazole could be selectively regulated by changing the amount of cuprous chloride. This method featured high regioselectivity, good functional group tolerance, and a wide substrate scope (Scheme 3D).

The possible reaction mechanism of these reactions is shown in Scheme 3E. AgN_3_ was prepared based on anion interchange of TMSN_3_ with Ag_2_SO_4_ and AgOH in the wake of the liberation of (TMS)_2_SO_4_ and TMSOH in a basic solution. Then vinyl azide 2 was obtained because of AgN_3_ insertion into the C-C triple bond *via* protodemetalation of vinylsilver 1 with H_2_O. Then 2 underwent 1,3-dipolar cycloaddition with the tautomerization of 1,3-dicarbonyl compound in basic conditions, which generated the coupling adduct 3. Finally, the triazole products were passed through the dehydration-driven aromatization process.

**SCHEME 4:**
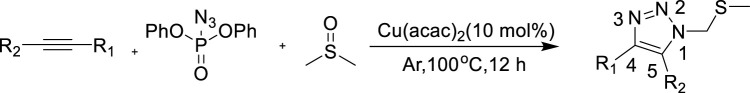
Synthesis of 1,4,5-trisubstituted-1,2,3-triazoles.

In 2015, Ning Jiao described an effective Cu-catalyzed nitrogenation of alkynes and alkenes for the rapid synthesis of sulfur-containing triazoles (Shen et al., 2015). Available sulfoxides and azides were employed as the S- and N-source to readily prepare highly value N- and S-containing 1,2,3-trizoles. A direct and practical solution was provided to gain sulfur-containing triazoles in this method (Scheme 4).

### Alkyl/Aryl azido as nitrogen source

**SCHEME 5:**
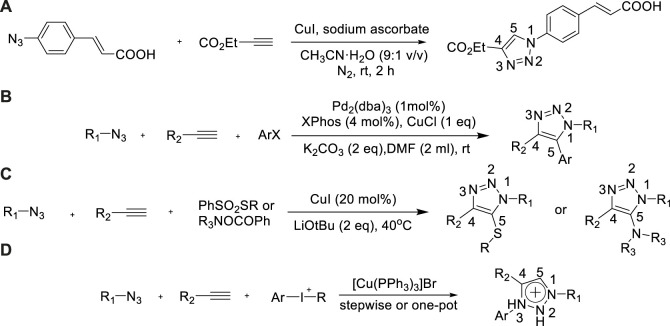
Pathways of synthesized triazole derivatives from azide.

In 2009, a simple method for the synthesis of 1,4-disubstituted-1,2,3-triazole from ethyl propionate was developed by Zhang et al. (2009). Sodium ascorbate and CuI were added to the suspension of 3-(4-azolophenyl) acrylic acid in CH_3_CN/H_2_O (9:1V/V) and stirred for 120 min under nitrogen atmosphere at air. 1-[4-(2-carboxyl vinyl) phenyl]-1,2,3-triazole-4-carboxylic acid ethyl ester could be prepared with high yield (90%)(Zhang et al., 2009) (Scheme 5A).

The substrates were large expanded based on aforementioned experiment. In 2015, the approach was reported by Zhenghu Xu which involved the production of multiple types of 1,4,5-trisubstituted-1,2,3-triazoles by Cu/Pd-catalyzed azide, alkyne, and aryl halide three-component reactions (Wei et al., 2015). 1,4,5-Trisubstituted-1,2,3-triazoles were assembled rapidly in one step with high yield (88%–98%)(Wei et al., 2015) and good regioselectivity using this route, which made up for the limitation of the traditional method CuAAC which was only suitable for the synthesis of triazoles from terminal alkynes (Scheme 5B).

In the following year, Zhenghu Xu further developed the method to construct N-alkyl-5-sulfur/selenium/amino-1,2,3-triazoles *via* using Cu-catalyzed alkyne, alkyl azide, and thiosulfonate or selenesulfonic ester (Wang et al., 2016). The reaction was reacted under mild conditions, with a broad scope of substrates, and various fatty mercaptols and aromatic thiophenols were well compatible with the conditions (Scheme 5C).

In 2019, Miha Virant et al. described an effective synthesis of 1,3,4-trisubstituted-1,2,3-triazole involving alkyne, azide, and aryl iodonium salt with the yield of 83% (Virant and Kosmrlj, 2019). The reaction had the advantages of simple operation and broad scope of substrates, which supplied a robust, selective, and effective method for the synthesis of various bioactive 1,3,4-triaryl-1,2,3-triazolium salts (Scheme 5D).

**SCHEME 6:**
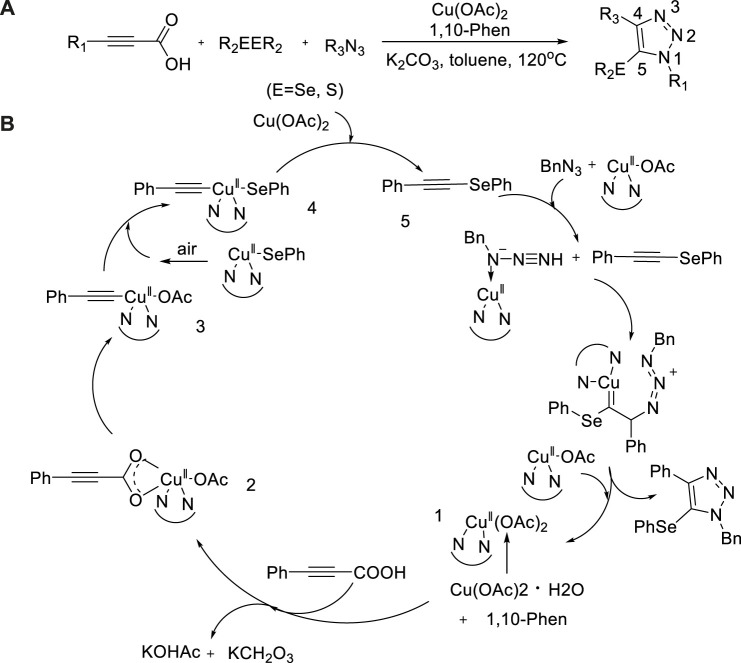
**(A)** Synthesis of N-alkyl-5-arylselanyl-1,2,3-triazoles. **(B)** Plausible mechanism of synthesize N-alkyl-5-seleno-1,2,3-triazoles.

In 2018, a copper-catalyzed three-component reaction of alkynic acid, diesel ether, and azide to produce N-alkyl-5-seleno-1,2,3-triazoles was described by Wang et al. (2018). Using copper acetate as a catalyst, N-alkyl-5-seleno-1,2,3-triazolium compounds could be obtained efficiently at 120°C. The reaction has the characteristics of simple operation, high regioselectivity, and good compatibility under air. A variety of N-alkyl-5-seleno-1,2,3-triazole compounds have been synthesized to show effective anticancer activity *in vitro* (Scheme 6A).

A possible mechanism is described in Scheme 6B. First, 1,10-phenanthroline reacted with Cu(OAc)_2_·H_2_O to form the active Cu(II) intermediate 1 that then reacted with phenylacetic acid, further underwent decarboxylation, and CO_2_ was released to form 3. Subsequently, 4 reacting with diphenyl diselenide following reductive elimination to generate phenyl(phenylethynyl)selane 5, as well as copper(I) species. The complexation of copper intermediate with phenyl(phenylethynyl)-selane and -azide, which underwent oxidative cyclization, turned into products under air atmosphere to finish the reaction.

**SCHEME 7:**
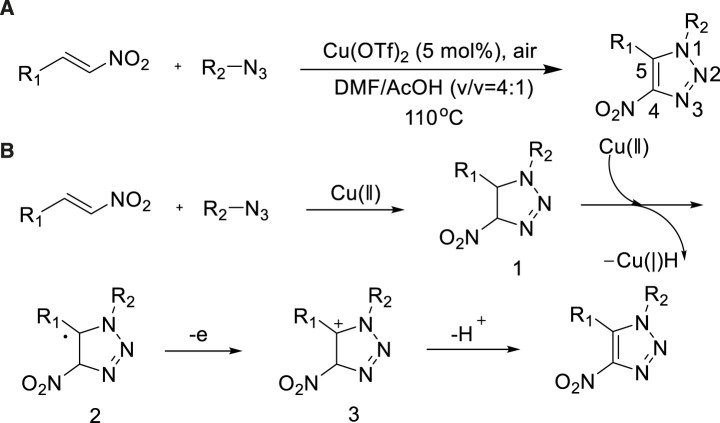
**(A)** Synthesis of 4-NO_2_-1,5-trisubstituted-1,2,3-triazoles. **(B)** Plausible mechanism for synthesis of 4-NO_2_-1,5-trisubstituted-1,2,3-triazoles.

In 2015, Chen et al. reported the synthesized method of 4-NO_2_-1,5-trisubstituted-1,2,3-triazoles from nitroolefins and organic azides by copper-catalyzed [3+2] cycloaddition or oxidation reaction (Chen et al., 2015). At 110°C, 4-NO_2_-1,5-trisubstituted-1,2,3-triazoles were prepared at high yield (96%) (Chen et al., 2015). The reaction was simple to operate, and the substrate was suitable for a wide range, which provided an effective route for the synthesis of 1,2,3-triazoles (Scheme 7A).

A probable mechanism of copper-catalyzed [3+2] cycloaddition or oxidative reaction is proposed in Scheme 7B. The regioselective 1,3-dipolar cycloaddition took place between nitro-olefin and azide to form the triazole intermediate 1; transition metal complexes could promote this process. Then 2 is obtained through Cu(II) catalyst, which could be stabilized with the help of an Ar group. The cation intermediate 3 was given because of this the radical lost another electron; then, 4-NO2-1,5-trisubstituted-1,2,3-triazoles would be formed *via* cation intermediate 3 losing a proton. In this reaction, The Cu(II) as a catalyst play an important role, which could be regenerated in acidic condition from Cu(I) species with O_2_.

**SCHEME 8:**
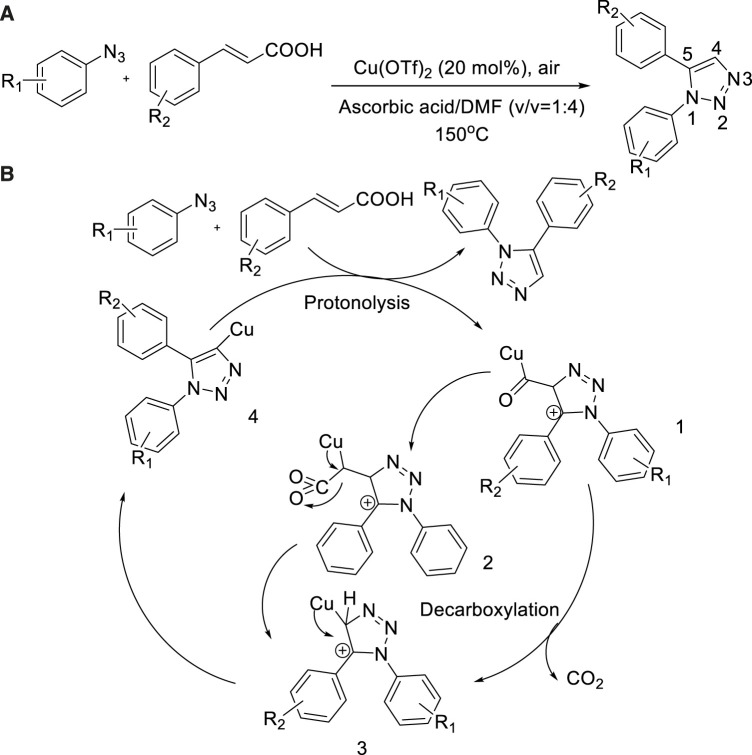
**(A)** Synthesis of 1,5-disubstituted-1,2,3-triazoles. **(B)** Plausible mechanism for Cu(II)-catalyzed 1,5-disubstituted-1,2,3-triazoles synthesis.

In 2018, Atul Kumar et al. showed that Cu-catalyzed decarboxylation of cinnamic acid with aryl azide to construct 1,5-disubstituted-triazoles (Kumar et al., 2018). Using Cu(OTf)_2_ as a catalyst and ascorbic acid/DMF as a solvent, 1,5-disubstituted-1,2,3-triazoles were obtained in good yield (80%) (Kumar et al., 2018) at 115°C. The adaptability of various aryl-substituted azides and cinnamic acid substrates was investigated, and it was found that various functional groups including halogen, nitro group, and cyanide group could be applied under the reaction conditions (Scheme 8A).

The proposed mechanistic pathway is shown in Scheme 8B: First, the regioselective 1,3-dipolar cycloaddition arose with azides and cinnamic acid to form the cation intermediate 1. Subsequently, 2 got passed decarboxylation to form copper triazoline 3 and then copper complex 1,4,5-trisubstituted-1,2,3-triazoles 4 were received because intermediate 3 lost a proton. The free 1,5-disubstituted-1,2,3-triazoles were provided due to 4 was easily undergone protonolysis. Also, it is important that this pathway allowed the Cu(II) species to rise again under the acidic media.

### Hydrazone as nitrogen source

In recent years, a method of synthesis N-substituted-1,2,3-triazoles from hydrazones instead of azides had been developed. In addition, 30 years ago, Sakai developed a fascinating scheme for the production of 1,2,3-triazole *via* the condensation of an amine and α, α-dichlorotosyl hydrazone in mild reaction conditions. The approach does not need a metal catalyst and azide, but this pathway had not been used adopted widely for a long period (Sakai et al., 1986).

**SCHEME 9:**
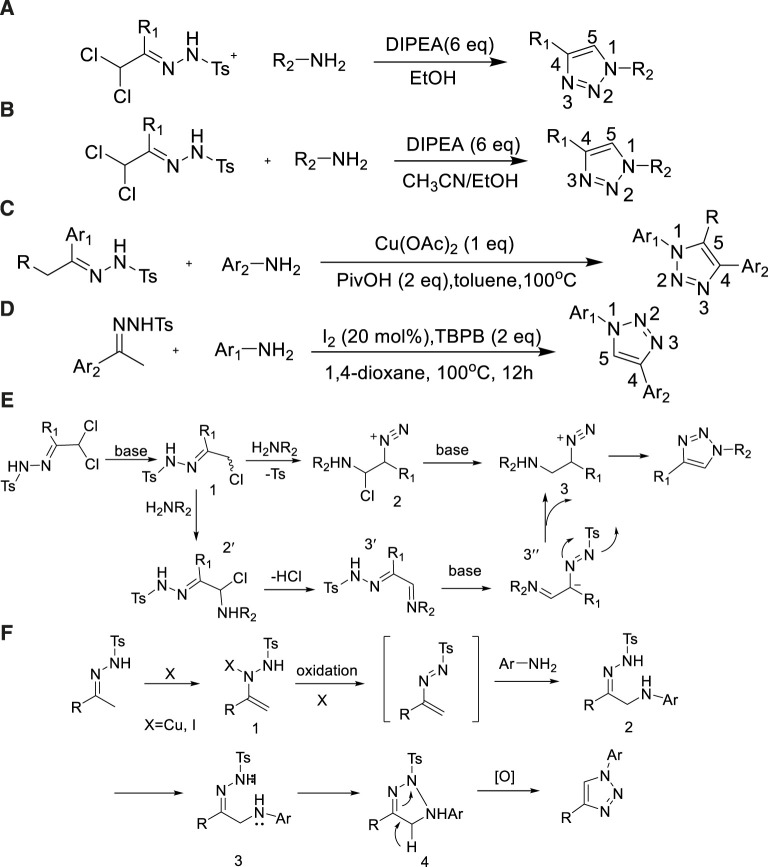
Synthesis of N-substituted-1,2,3-triazole framework and the mechanism of N-substituted-1,2,3-triazole framework.

In 2010, Hanselmann further applied the cyclization reaction of α, α-dichlorotoluene sulfonyl–substituted hydrazone with primary amine mediated by DIPEA to synthesize N1-substituted-1,2,3-triazoles (Roger Hanselmann et al., 2010). N-substituted-1,2,3-triazoles with antibacterial activity can be synthesized in an ethanol solvent by this method. The key 1,2,3-triazole skeletons could be easy obtained under the presence of DIPEA on a large scale with the yield of 80% (Roger Hanselmann et al., 2010), which avoided potential safety concerns (Scheme 9A).

Subsequently, Berkel et al. improved the reaction conditions by adopting a mixed solvent of acetonitrile and ethanol to broaden the range of reaction substrates (van Berkel et al., 2012) (Scheme 9B).

In 2013, Zhang showed the method to synthesis 1,4,5-trisubstituted-1,2,3-triazoles by copper-mediated reaction of N-tosylhydrazones and aniline with the yield of 82% (Chen et al., 2013). Significant features of this scheme involved the unique constituent of 1,4- or 1,4,5-substituted regioisomers, the advantages of wide scope with regard to low cost of reagents, and the convenient operating conditions for the N-tosylhydrazones and the aniline substrates (Scheme 9C).

In 2014, an efficient method for the synthesis of 1,4-diaryl-1,2,3-triazoles from N-tosylhydrazones with anilines under the mediation of I_2_/TBPB (terbutyl benzoate peroxide) system was reported by Cai et al. (2014). The method used I_2_ as a catalyst and TBPB as an oxidant without any metal catalyst. A variety of 1,4-diaryl-1,2,3-triazoles could be produced with moderate to good yields (89%) (Chen et al., 2013) (Scheme 9D).

The general mechanism of Scheme 9A,B was depicted as Scheme 9E. First, α, α-dichlorotosyl hydrazone was converted into vinyldiazine 1 that passed the process of elimination of HCl; intermediate 2 was formed *via* the amine addition and toluenesulfinate ion elimination. Finally, 1,2,3-triazoles were produced through deprotonation and cyclization.

The preliminary mechanism of Scheme 9C,D was depicted as Scheme 9F. The N-tosylhydrazone might isomerize to 1. Subsequently, 1-tosyl-2-vinyldiazene 2 was given *via* N-tosylhydrazone oxidation and then the intermediate 3 was produced by an aza-Michael addition with aniline. Finally, the 1,2,3-triazole derivatives were prepared through the Cu/I-catalyzed cyclization of 3 with the formation of a N-N bond and subsequent aromatization. A radical process occurred in the reaction.

**SCHEME 10:**
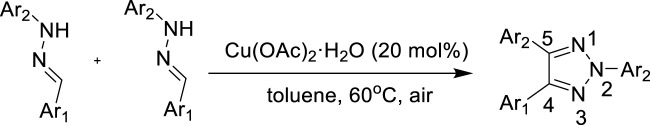
Synthesis of 2,4,5-triaryl-1,2,3-triazoles.

In 2012, Guru developed a copper-catalyzed production of 2,4,5-triaryl-1,2,3-triazoles from bis(arylhydrazones) under a mild condition (Guru and Punniyamurthy, 2012). In this method, a variety of N_2_-aryl-1,2,3-triazoles were produced by continuous C-H functionalization and C-C, N-N, and C-N bond formation reactions with Cu(OAc)_2_ as a catalyst. This scheme took place under mild reaction conditions, could be utilized in large synthesis range, and possessed wide functional compatibility with easily available substrates (Scheme 10).

### Hydrazine as nitrogen source

Hydrazine, as an important chemical raw material, is often used to construct various nitrogen-containing compounds.

**SCHEME 11:**
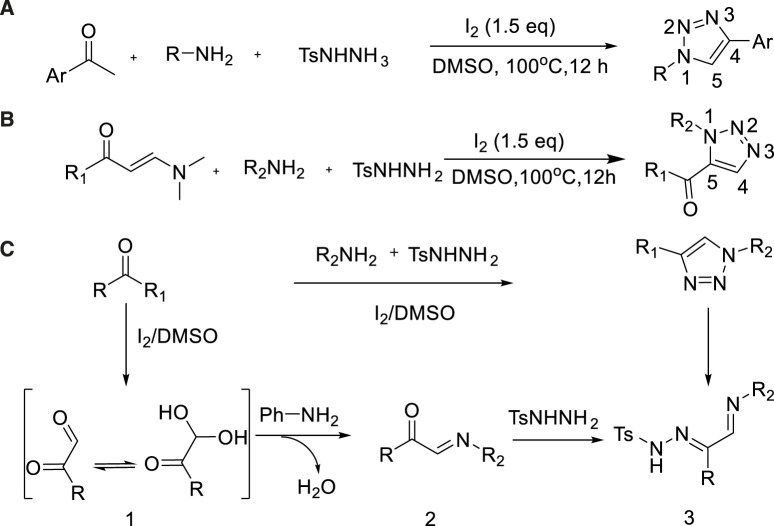
Synthesis and mechanism 1,4-disubstituted-1,2,3-triazoles.

In 2014, Zhang et al. reported the nonmetal-mediated synthesis of 1,4-disubstituted-1,2,3-triazole by the three-component reaction of aniline, aromatic ketone, and 4-methylbenzenesulfonohydrazide (Chen et al., 2014). In this method, various types of 1,4-disubstituted-1,2,3-triazoles were prepared which exhibited good to excellent yield (75%–92%)(Chen et al., 2014) by successive formation of C-N and N-N bonds. The reaction condition was mild and did not require metal reagent and azide providing nitrogen atom. (Scheme 11A).

In 2015, Wan Jiping expanded the scope of substrate, developed a method to construct 1,2,3-triazoles from enamine–ketone, primary amine and p-toluene sulfonyl hydrazine catalyzed by non-metallic iodine (Wan et al., 2015). The strategy was accomplished by continuous C-N bond and N-N bond formation and acyl migration, and multiple N1-substituted-1,2,3-triazoles were synthesized with good yields (78%)(Wan et al., 2015) (Scheme 11B).

A plausible pathway is proposed (Scheme 11C). Phenylglyoxal intermediate 1 was created by Kornblum oxidation with the aniline in the presence of iodine and DMSO. The 1 passed through condensation with the aniline to obtain the C-acyl imine intermediate 2, which passed by condensation with N-tosylhydrazine formed 3. Subsequently, the triazole products were obtained by intermediate 3 out of cyclization and aromatization under molecular iodine or O_2_.

### Diazo compounds as nitrogen source

**SCHEME 12:**
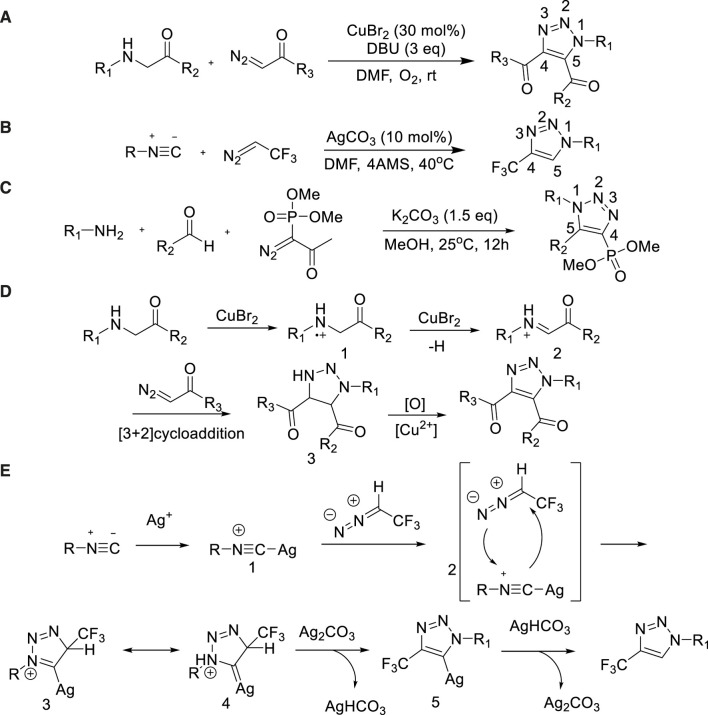
Method of synthesizing N-substituted-1,2,3-triazole by [3+2] cycloaddition reaction and the possible mechanism.

In 2015, a series of N1-substituted-1,2,3-triazoles were prepared through Cu-catalyzed [3+2] cycloaddition reaction of secondary amines and diazo compounds by Li et al. (Li et al., 2015). This reaction used oxygen as a green oxidant and had the advantages of easy availability of raw materials, mild conditions and good compatibility of functional groups. It provided a practical synthesis method for a series of N1-substituted-1,2,3-triazoles in 55%–84% yields (Li et al., 2015) (Scheme 12A).

In 2015, Ma et al. reported a similar convenient method based on Ag-catalyzed cycloaddition reaction of isonitrile and diazide [3+2] to prepared a series of N1-substituted-1,2,3-triazoles (Wang et al., 2015) (Scheme 12B).

In 2016, Mohanan developed a metal-free, effective and easy way based on three-component reaction for the synthesis of 1,4,5-trisubstituted-1,2,3-triazoles (Ahamad et al., 2016). Using cheap and readily available aldehydes, amines and Bestmann Ohira reagents (BOR) as feedstock, the reaction provided an efficient synthesis strategy for 1,4,5-trisubstituted-1,2,3-triazoles with yield from 47% to 63% (Ahamad et al., 2016). Took into consideration the relevance of triazole scaffolds in the agrochemical and pharmaceutical, adopting this scheme to synthesize phosphonyl triazoles would be found potential applicability (Scheme 12C).

The mechanism of Scheme 12C was similar as Scheme 12(a) were described in Scheme 12D: The first step, amine was oxidized by CuBr_2_ passed electron transfer process to form amine radical cations 1. Then hydrogen radicals were transferred to form imine cations 2, which performed the [3+2] cycloaddition reaction with α-diazo compounds to obtain 3. Finally, three was oxidized by CuBr_2_ in the presence of oxygen to form N1-substituted-1,2,3-triazole.

The mechanism of Scheme 12B was described in the Scheme 12E: Firstly, isonitrile reacted with Ag + to produce silver isonitrile compound 1. And then, 1 with 2,2,2-trifluoromethyl diazomethane underwent the 1,3-dipolar cycloaddition reaction, following isomerization to obtain silver carbine compound 4 and then deprotonated. Finally, the silver was removed by hydrogenation reduction to form N1-substituted-1,2,3-triazole.

### Synthesis of 1,2,4-triazoles

1,2,4-triazole is an important heterocyclic motif, which widely existed in the molecular structures with a sequence of bioactivities, including anticancer, antibacterial, anti-HIV and so on. The main synthesis methods include amidines, imidates, amidrazones, aryldiazoniums and hydrazones as raw material to provide nitrogen atoms to synthesize 1,2,4-triazole derivatives.

### Amidines as nitrogen source

**SCHEME 13:**
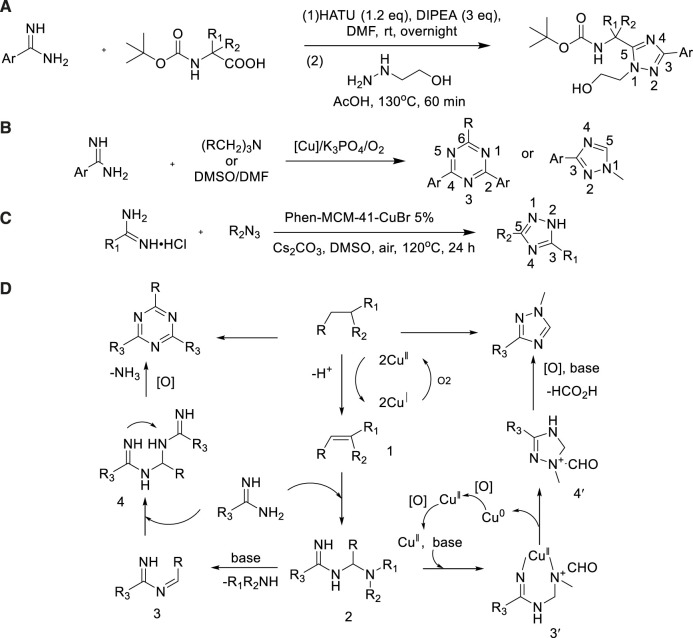
Synthesis and mechanism of N-disubstituted-1,2,4-triazole derivatives.

Amidine was widely used as an organic catalyst and ligand for the formation of nitrogen–carbon bonds due to the reactivity of nucleophilic nitrogen atoms. In 2011, a general method had been developed *via* one-pot, two-step process for the production of 1,3,5-trisubstituted-1,2,4-triazole with excellent yield (up to 90%) (Castanedo et al., 2011). The sequence started with the *in situ* formation of amide from carboxylic acid and amidine. Aniline further reacted with monosubstituted hydrazine and cyclized to trisubstituted triazole. This method had the advantages of high regioselectivity and good tolerance of functional groups (Scheme 13A).

In 2015, Huang described an efficient CuCl_2_ promoted synthesis of 2,4,6-trisubstituted and 1,3-disubstituted-1,2,4-triazoles (Huang et al., 2015). Triazole was prepared with high yield (85%) (Huang et al., 2015)under the presence of CuCl_2_ and DMF using K_3_PO_4_ as alkali, O_2_ as oxidant, amide and DMF as raw materials. In fact, nitrogen atoms and methyl groups from DMF were incorporated into the triazole. For amides replaced by various functional aromatic fragments (fluorine, bromine, methyl, methoxy, and trifluoromethyl), the reaction sequence appears to be highly effective (scheme 13B).

In 2019, Xia developed a facile copper-catalyzed one-pot method to prepare 3,5-disubstituted-1,2,4-triazole from amide and nitrile by cascade addition oxidation cyclization (Xia et al., 2019). In this reaction, O_2_ was used as an oxidant, and functionalization was catalyzed by the complex of McM-41 and cuprous bromide [phen-McM-41-CuBr] with high yield (91%) (Huang et al., 2015). The pathway was utilized to prepare a various of 1,2,4-triazole derivatives (Scheme 13C).

The possible reaction mechanism shown in Scheme 13D. As 2) showed Cu-catalyzed C (sp3)-H firing the amine to prepare imine-type intermediate 1 *via* a single-electron transfer (SET) procedure would be the first step of this reaction. In the following process, nucleophilic of 1 attack by the amidine to form intermediate 2. Taking the case of tertiary amine, the amine motif was delivered to get imine intermediate 3. Another molecular amidine 4 undertook the second attack of 3 with an intramolecular nucleophilic cyclization and followed oxidation to generate the final 1,3,5-triazine compounds.

### Imidates as nitrogen source

**SCHEME 14:**
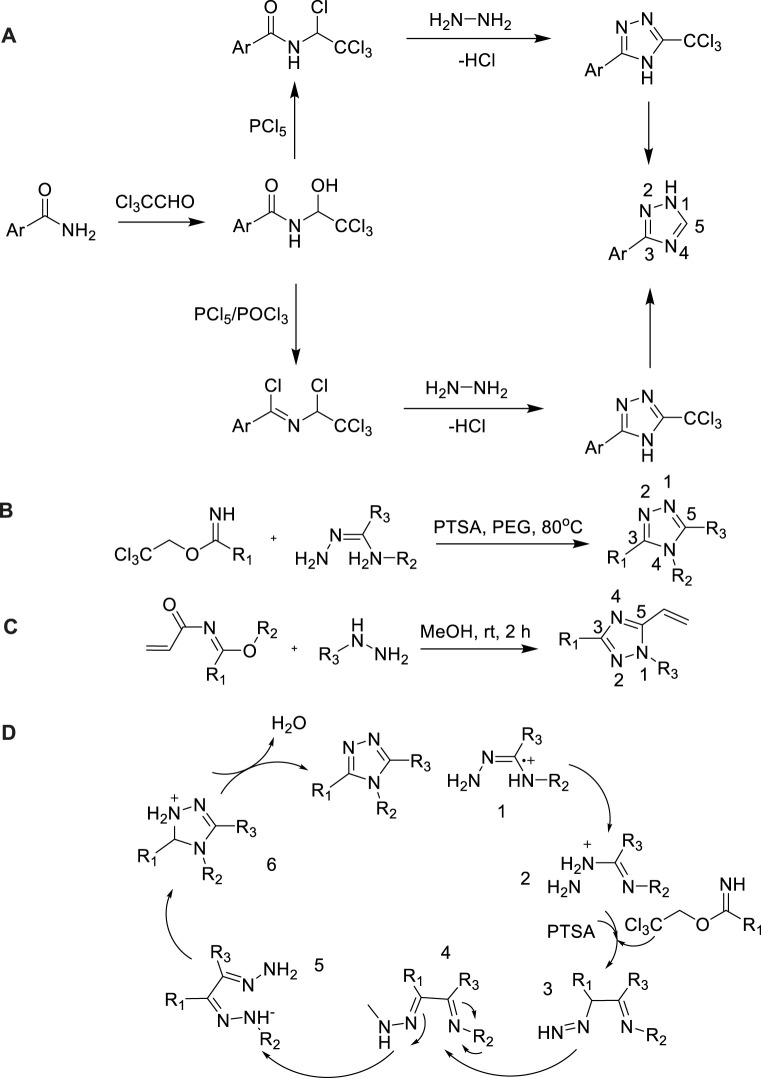
**(A)** Synthesis of 3-aryl-1,2,4-triazole. **(B,C,D)** Synthesis and mechanism of 3,4,5-trisubstituted-1,2,4-triazoles.

Imidates are helpful precursors of triazoles, which have been extensive wielded in this field. In 2016, Guirado proposed a simplified method for the synthesis of 3-aryl-1,2,4-triazole chloroformamide, which was obtained *via* the reaction of benzamide with chloral hydrate in high yield (Guirado et al., 2016). When imidates reacted with the mixture of phosphorus pentachloride/phosphorus oxychloride, almost quantitatively converted to bis(1-tetrachloroethyl) benzimine chloride, which was then handled with hydrazine hydrate to produce 3-aryl-1,2,4-triazoles with high yield (86%) (Huang et al., 2015). The method had the advantages of good versatility, high yield, easy access to starting materials, mild and simple experimental steps (Scheme 14A).

In 2014, Mangarao et al. had prepared a series of 3,4,5-trisubstituted-1,2,4-triazoles from 2,2,2-trichloroethyl imidate, which employed polyethylene glycol (PEG) as a solvent and used p-Toluenesulfonic acid (PTSA) as the catalyst during mild conditions (Mangarao et al., 2014). In conclusion, this was a facile, effective, and ecofriendly simple method for the synthesis of 1,2,4-triazole from 2,2,2-trichloroethyl imidate in PEG and employing PTSA as the catalyst leading to the corresponding 1,2,4-triazoles in mild conditions with excellent yields (92%) (Mangarao et al., 2014) (Scheme 14B).

In 2018, Azzouni et al. focued on the production of vinylimidates as precursors for the synthesis of functionalized 1,2,4-triazoles. (Azzouni et al., 2018). The method allowed a large array of substituents to be installed at (N1, C3, and C5) positions of 1,2,4-triazole with good yield (98%) (Azzouni et al., 2018). The experiment showed that the selectivity formation of 5-vinyl-1,2,4-triazoles could arrest take place potential five-member and seven-member by-products *via* theoretical calculations and NMR (Scheme 14C).

Take Scheme 14B as an example, the possible reaction mechanism described in Scheme 14D. PTSA catalyzed 2,2,2-trichloroethyl imidates undergone a series of oxidate and cyclization reaction to generate 1,2,4-triazole derivatives. PTSA played a significant role in this reaction. PTSA was not only a catalyst, but also a Lewis acid participated in reaction.

### Amidrazones as nitrogen source

**SCHEME 15:**
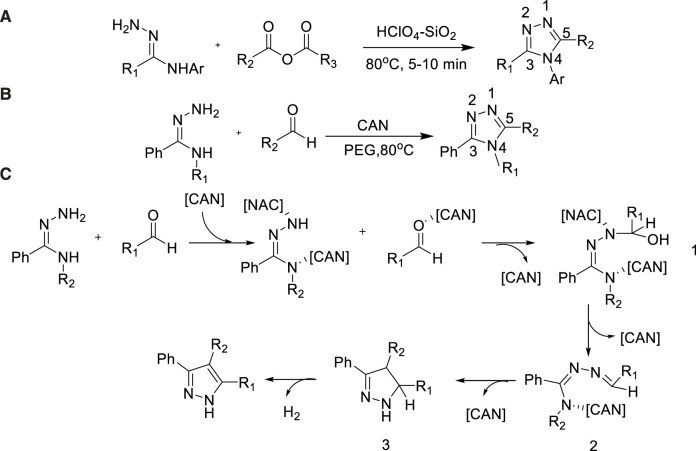
Synthesis and mechanism of 3,4,5-trisubstituted-1,2,4-triazoles.

Amidine was an excellent raw material to provide nitrogen atom in the synthesis of triazole. In 2011, Siddaiah et al. reported that 1,2,4-triazole derivatives can be prepared at 80°C with moderate to high yields (55%–95%) in solvent-free conditions using HClO_4_-SiO_2_ as a catalyst (Siddaiah et al., 2011). The advantages were that the substituents on amide hydrazone and anhydride had good tolerance during optimized conditions, and the HClO_4_-SiO_2_ catalyst could be recycled continuously for at least three times (Scheme 15A).

In 2014, Vidavalur et al. developed a similar method *via* using ceric ammonium nitrate (CAN) oxidative cyclization synthesis of 3,4,5-trisubstituted-1,2,4-triazole in polyethylene glycol (PEG) with good to excellent yield from 61% to 97% (Vidavalur et al., 2014). In this reaction, CAN was not only an oxidant, but also a Lewis acid. After the activation of amino nitrogen atom, benzaldehyde was added to generate diazizone intermediate. Finally, oxidation cyclization reaction was carried out to synthesize 1-(sulfur)-phosphonated 5-amino-1,2,4-triazole framework. The reaction conditions were relatively easy to operate (Scheme 15B).

A possible mechanism was showed in Scheme 15C. Ceric ammonium nitrate reacted as both a Lewis acid and an oxidant. CAN play an irreplaceable role in promoting the occurrence of the reaction.

**SCHEME 16:**
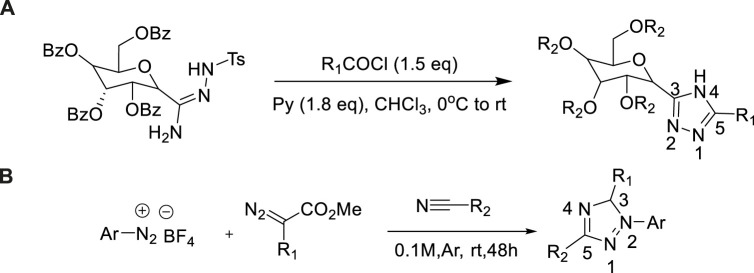
Synthesis of C-Glucopyranosyl-1,2,4-triazoles and 2-aryl-3,5-disubstituted-1,2,4-triazole derivatives.

In 2013, the synthesis of 3-(β-d-Glucopyranosyl)-5–substituted 1,2,4-triazoles from c-glucose-toluene sulfonyl hydrazone was reported by Bokor et al. (Bokor et al., 2013). 3-(β-d-Glucopyranosyl)-5-substituted-1,2,4-triazoles were constructed by acylation of O-perbenzoylated N1-tosyl-C-β-d-glucopyranosyl formamidrazone and subsequent removed protecting groups. The acylation of C-glucosyl toluene sulfonyl hydrazone with aromatic acid chloride was accompanied by a complete N-decarboxylation reaction, yielding a high yield (62%–92%) (Azzouni et al., 2018) of 3-(B-d-glucosyl)-5-substituted-1,2,4-triazole. This series of compounds were evaluated as effective glycogen phosphorylase inhibitors, which extended the substrate of 1,2,4-triazole framework construction (Scheme 16A).

In 2017, Benassi et al. composed a variety of 4,5-dihydro-1H-1,2,4-triazole from various amide hydrazones (Eliseeva et al., 2017). In the presence of pyridine in toluene, the group 1,2,4-triazole was firstly synthesized from amide hydrazone and dimethyl or diethyl ester to obtain the corresponding 2-aryl-3,5-disubstituted-1,2,4-triazole. Under microwave irradiation, aminolazone reacted with excess acetone to produce 5,5-dimethyl-4,5-dihydro-1,1,2,4-triazole compounds. Photophysical result indicated that most of the 1,2,4-triazole compounds with blue or yellow-green luminescence, and the fluorescence emission ability was medium, which had a good application value in the preparation of fluorescent probes (Motornov et al., 2018) (Scheme 16B).

### Aryl diazonium salts as nitrogen source

**SCHEME 17:**
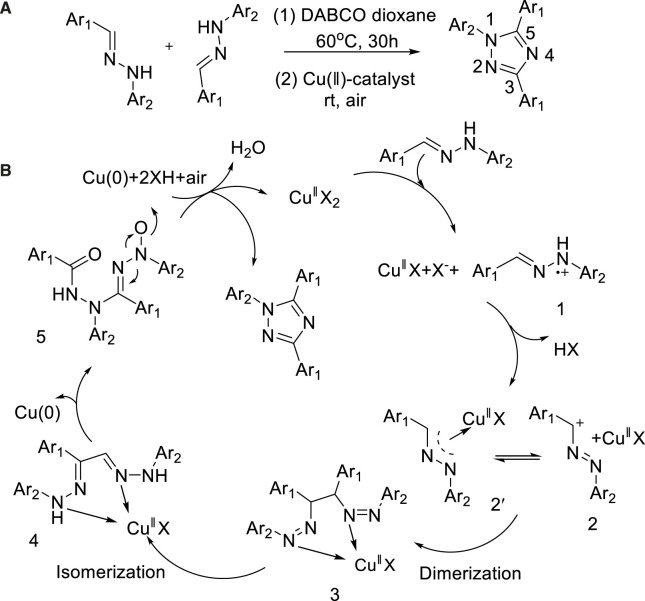
Synthesis and mechanism of 1,2,4-triazole.

Aryl diazonium salts as intermediates, which were utilized broadly as nitrogen source for the generation of abundant heterocycles compounds due to the advantages of shortcutting available and highly reactive. In 2020, Tian et al. developed the synthesis of 1,2,4-triazole by decarboxylation and cyclization of 2-aryl-2-isocyanate with aryl diazonium salts (Tian et al., 2020). In this metal-free reaction, the use of 1,4-diazocyclic [2.2.2]octane (DABCO) as a weak base was extremely critical (Scheme 17A).

A possible mechanism was described in Scheme 17B. Thus, bis(arylhydrazones) could be converted into the radical cation 1 *via* single-electron transfer (SET) to Cu(II)X2. Base on the elimination of HX, that intermediate 1 could be transformed to 2. 2 could lead to the formation of 3 pass by dimerization, which could isomerize to form the intermediate 4. The latter could afford a radical cation 5 by reductive elimination of elemental copper through SET process. The catalytic cycle might be completed *via* regenerate the active Cu(II) species from reduced Cu(0) species was reoxidized by air. This was conducive to promoting the process of response. Besides, DABCO is not only acting as a base but also playing a crucial role of an effective ligand for the Cu(II)-catalyzed oxidative cyclization.

**SCHEME 18:**
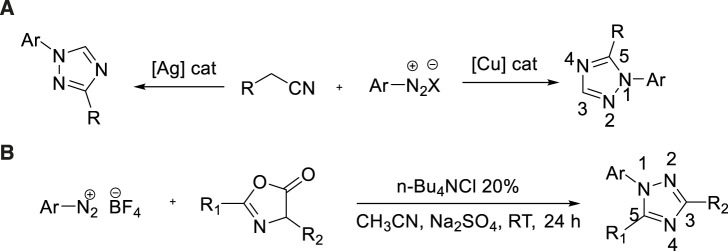
Synthesis of 1,3- and 1,5-disubstituted-1,2,4-triazoles.

In 2018, a highly effective method for the production of 1,5-disubsubstituted-1,2,4-triazole was reported by Liu et al. (Liu et al., 2018). According to the properties of catalytic system, triazole was synthesized by aryl diazonium salts and isocyanide [3+2] cycloaddition method. In fact, the utilization of Ag(I) as a catalyst produced selective 1,3-disubstituted-1,2,4-triazole in the yield of 88%, while the copper catalyst produced 1,5-disubstituted-1,2,4-triazole in the yield of 79% (Scheme 18A).

In 2019, Yu et al. firstly studied metal-free [3+2] cycloaddition/decarboxylation between aryl diazonium salts and azalactic acid to obtain 1,3,5-trisubstituted-1,2,4-triazole (Yu et al., 2019). The optimized reaction conditions were as follows: 20mol% N-Bu_4_NCl and Na_2_SO_4_ (as desiccant) in acetonitrile at room temperature. N-Bu_4_NCl could be used as a source of chlorine, which could stabilize the intermediate in the cycloaddition reaction of aryl diazo and enol type azalactone [3+2]. The intermediate was then subjected to ring-opening decarboxylation to form a 1,2,4-triazole target. It was a feasible synthesis of 1,2,4-triazole derivatives (Scheme 18B).

### Hydrazones as nitrogen source

**SCHEME 19:**
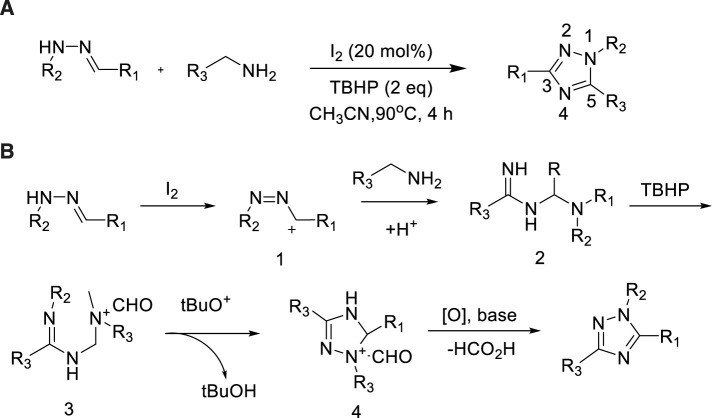
I_2_-catalyzed synthesis of 1,2,4-triazoles and possible mechanism.

Hydrazones were popular precursors of nitrogen heterocycles in most methods for the production of fused 1,2,4-triazole. Characteristics of these compound involved highly reactive nitrogen pairs and SP-carbon atoms. Hydrazones could react with various amines to synthesize triazoles. In 2016, Chen et al. developed a synthetic method in which hydrazone could be easily converted to 1,2,4-triazole by combining amines with I_2_/TBHP in high yield (92%)(Chen et al., 2016). A wide range of functional groups were well tolerated in hydrazone and amine fragments for the synthesis of multiple compounds. The method has the characteristics of available raw materials, simple and easy operating conditions, a wide range of substrates (Scheme 19A).

A possible mechanism of the framework was presented in Scheme 19B. The Michael-type nucleophilic supplement of the terminal amino group of the benzamidrazone to form the malononitrile receptors 2. Then, the homologous spirocyclic 1,2,4-triazoles finished cyclization undergone an intramolecular nucleophilic supplement of the amino group to the imidic carbon atom to afford final 1,2,4-triazole derivatives.

**SCHEME 20:**
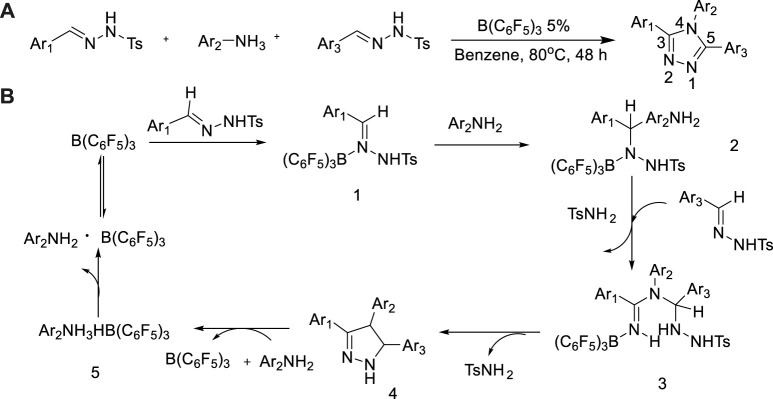
**(A)** Access to 3,4,5-trisubstituted-1,2,4-triazoles from N-tosylhydrazones and aromatic amines. **(B)** Plausible mechanism base on experimental.

In 2019, Guru et al. described a metal-free catalytic method for dehydrogenation cyclization based on B(C_6_F_5_)_3_ (Guru et al., 2019). B(C_6_F_5_)_3_ activated the hydrazine portion nucleophilic attack in progress, following in proper order amination, intramolecular cyclization and dehydrogenation steps to prepare the 3,4,5-tsubstituted-1,2,4-triazole with 85% yield (Guru et al., 2019). This reaction route was characterized by green economy, no oxidant, mild conditions and selectivity, which provided a potential platform for catalytic chemical conversion without the use of transition metal (Scheme 20A).

A predicted mechanism was described in Scheme 20B (Kojima, 2016; Cai et al., 2014). On account of recommended mechanism of the B(C6F5)3-catalyzed receptors less dehydrogenative cyclization of N-tosyl-hydrazones with anilines, investigators had carried out DFT calculations to research the sophisticated reaction mechanism and to receive deep understanding of driving force for the production of the 1,2,4-triazoles. The experimental calculations results revealed that the rate-determining step involved intra-molecular hydrogen transfer between the N-centers after aniline gets bonded to the N-tosylhydrazone unit.

**SCHEME 21:**
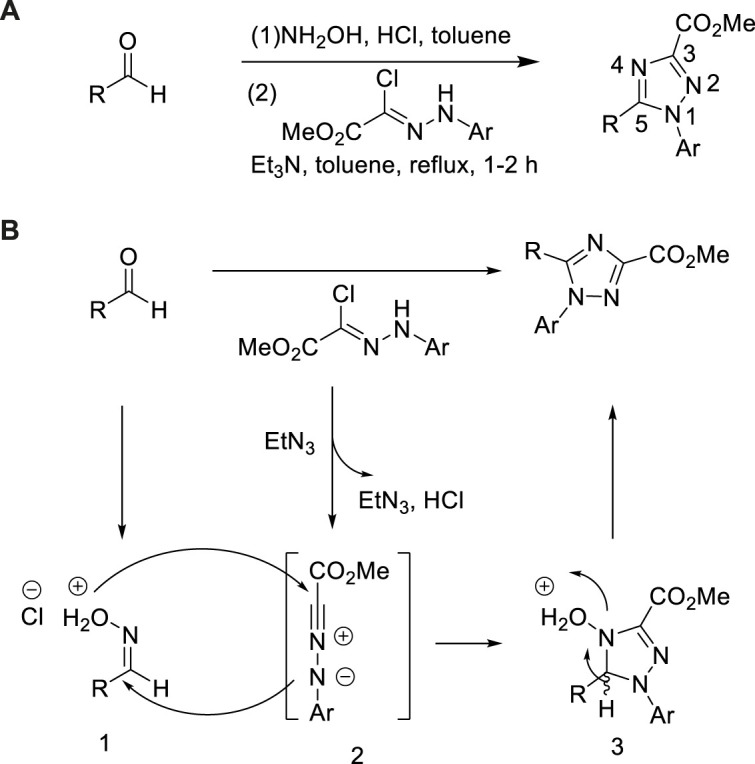
Synthesis and mechanism of 3,5-disubstituted-1,2,4-triazole.

In 2011, Wang et al. developed the preparation of 1,3,5-trisubstituted-1,2,4-triazole from hydrazonoyl hydrochlorides and aldehyde (Tseng et al., 2011). In the presence of triethylamine, aromatic rings with various substituents were suitable for this method as a 1,3-dipolar nitroimide source. In this one-pot reaction, the aldehyde was firstly converted to an oxime intermediate by reacting with hydroxylamine. The resulting oxime and hydrazyl hydrochloric acid were reacted by 1,3-dipolar cycloaddition to obtain 3,5-dissubstituted 1,2,4-triazole with yields ranging from moderate to good (53%–91%) (Tseng et al., 2011). This method could be provided to a series of aldehydes including aliphatic, cyclic aliphatic, aromatic, and heterocyclic aldehydes (Scheme 21A).

The plausible mechanism was represented in Scheme 21B. Acetaldehyde was reacted with hydroxylamine hydrochloride in toluene under reflux to prepare oxime hydrochloride intermediate 1. Nitrilimine specie 2 was generated *via* deal with hydrazonoyl hydrochloride by a surfeit of triethylamine. The essential 1,3-dipolar cycloaddition dihydrotriazole three was constructed by disposing dipolarophile oxime with 1,3-dipole nitrilimine. When the subsequent dehydroxylation condensation was finished, the corresponding 1,3,5-trisubstituted-1,2,4-triazole product was produced.

**SCHEME 22:**
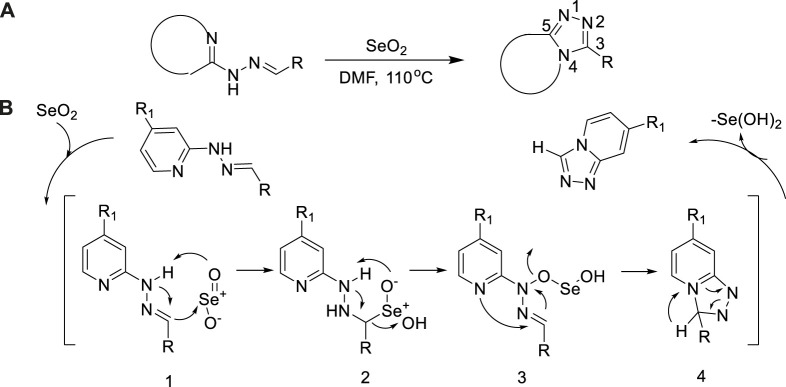
Selenium dioxide-mediated synthesis of 1,2,4-triazole and the speculative mechanism.

In 2015, Zheng et al. reported an efficient and actual methodology for synthesizing 1,2,4-triazoles by oxidative cyclization of hydrazones using SeO_2_ (Zheng et al., 2015)_._ A sequence of fused 1,2,4-triazoles were constructed by oxidative intramolecular cyclization between heterocyclic hydrazones and selenium dioxide. Simple applicability of this approach was corroborated base on these compounds of 1,2,4-triazolo [4,3-a] pyridines, 1,2,4-triazolo [4,3-a] pyrimidines, and 1,2,4-triazolo-[4,3-a] quinoxalines with moderate to good yield (79%–98%)(Zheng et al., 2015) (Scheme 22A).

The reaction mechanism may be through oxidative addition and reductive elimination processes (Scheme 22B). Hydrazones of aromatic and aliphatic aldehydes containing electron-withdrawing and electron-donating substituents were oxidized by SeO_2_, and then through a series of electron transfer, finally removed a molecule Se(OH)_2_ to give the corresponding 1,2,4-triazoles.

## Conclusion

In this review, we summarized the synthetic methods of 1,2,3-/1,2,4-triazoles combined with the progress of triazole over the past 2 decades. Several main synthetic methods from various nitrogen sources were summarized. The synthetic methods were sorted according to the key starting material utilized for the production of the 1,2,3-/1,2,4-triazole skeleton. Sodium azido, trimethylsilyl azido, alkyl/aryl azido, hydrazone, sulfonamide, hydrazine, and diazo compounds are among the most correlative 1,2,3-triazole precursors. Amidines, imidates, amidrazones, aryldiazoniums, and hydrazones are among the most correlative 1,2,4-triazole precursors. In the synthesis of 1,2,3-triazole, sodium azide is a highly toxic compound, which is not suitable for large-scale application as a nitrogen source. Compared with sodium azide, azide trimethylsilane and alkyl/aryl azide are safer and more efficient. Hydrazone, aminohydrazine and diazo compounds are also the main materials of nitrogen sources, which were reported in a large number of literatures. The synthesis method of 1,2,4-triazole mainly uses amidine, amide, amidine hydrazone, aryl diazonium salt, and hydrazone as nitrogen sources. During the past few years, there have been excellent progress in copper-catalyzed cross-couplings in the synthesis methods of 1,2,3/1,2,4-triazole with inexpensive, low toxic, and good functional tolerance. Metal catalysis was the mainly choice at present; among them, copper-catalyzed was the most popular catalyst. With the promotion of the concept of “green chemistry”, metal-free catalytic synthesis such as electrocatalysis and photocatalysis are attracting researcher’ interest. It is believed that with the continuous efforts of researchers, more green economy and simple and mild synthesis methods will continue to develop.
